# Mental Nurses in War Hospitals

**Published:** 1916-10-21

**Authors:** 


### THE EDITOR'S LETTER-BOX.

### Mental Nurses in War Hospitals.

*To the Editor of* The Hospital.

Sir,—The Editorial comment on the letter of "Justice" in The Hospital of August 26, p. 27, and that on "A Reply from a Correspondent" in the number of September 30, convey to some, at any rate, of those who are associated with the care and nursing of the insane the opinion that the author is either ignorant or misinformed as to the training and capabilities of those women who make mental nursing their profession.

As a correspondent states in his letter appearing in The Hospital of October 7, he has conceded nothing that was asserted in previous communications. There was nothing that could be conceded, but it seems only just that on behalf of the trained mental nurse something further should be said to prove that she is not a mere ignorant maid, and that the training she has to undergo is not a farce.

It is obvious that, in her letter, which has brought forth such comments, "Justice" merely asks that she and other trained mental nurses be recognised and allowed to help in war hospitals, and anyone who is cognisant with the capabilities of those nurses will admit that they would be most efficient helpers. She is not asking to be placed over the nurse trained in a general hospital, and the comment on her letter, disparaging as it is to the training, capabilities, and social status, is far from being temperate. No nurse trained in a mental hospital only would assert that she was the equal of her sister from the general hospital in surgical nursing. The former knows her limitations, but the same cannot always be said of the latter with regard to mental nursing. If, as is maintained, each must stick to her own last, why is it that nurses without mental training attend mental cases, and also in some cases are ready to take high posts in mental hospitals, thereby depriving the mental nurse of the fruits of years of strenuous learning in her calling? The fact that in so far as her work is confined to technical skill in dressing wounds the surgical nurse is superior to the mental nurse must be admitted, but when it comes down to general ward work, management, and discipline, the positions are equalised, if not reversed.

"Justice" in her letter states that the authorities connected with war hospitals do not seem to grasp what training a mental nurse has; it were well if she had gone further and stated this with regard to nursing authorities in general, excepting, of course, mental. It has been admitted that in technical skill the hospital nurse is in surgical work the superior of the trained mental nurse, but with regard to the nursing of medical cases there is little to choose between them. To say that there is shows ignorance of the training given in mental hospitals to the nurses, and incidentally of the work and objects of these institutions.

In the dark days when mental hospitals were known as "madhouses" and the inmates were mainly looked upon as unfortunates who had to be "put away," the statements made in the comments would hold good, but to-day the old order of things is changed. Those afflicted with disease of the mind are now looked upon as patients suffering from a definite form of disease requiring special treatment and careful nursing. The asylum has become hospitalised, and its main objectives are first and foremost cure, and, if this be not possible, alleviation for the sufferers who enter it. Such work cannot be carried out without nurses well trained medically as well as in mental diseases, and this training is given, and the nurses undergo it in the mental hospital. Also the nurses learn their work by practical experience in addition to the clinical demonstrations and lectures; nor is the result of such training a mere smattering of knowledge. Has the woman, trained in a mental hospital, who nurses as they should be nursed the acute mental states and many chronic conditions accompanied by marked physical disorder, not to mention the many purely bodily diseases which attack the insane just as they do the sane, a mere superficial idea of medical nursing? Most assuredly not. With regard to surgical nursing, though not a skilled expert, the trained mental nurse has sound knowledge of its principles, and this also learnt practically. True, major surgery in the mental hospital is rare, but minor cases are fairly common, from which much nursing knowledge can be gained.

However, whether nursing is divided into surgical or medical is not of great importance, but surely it will be admitted that the careful keeping of charts, the looking after the hygiene of the body, attention to the emunctories, administration of medicine, diets, to mention only a few duties of a nurse, and, above all, a keen power of observation which the mental nurse must especially acquire because the insane frequently cannot or will not complain, constitute nursing.

These facts are not stated with any object of putting the trained mental nurse in any but her true position, or to make her training out to be more comprehensive than it is, but merely to show that she is capable of nursing in all its aspects, and that her training is not all a farce.

As a correspondent of September 30 very truly states, the view that the work is less arduous and the discipline less strict in a mental hospital is untenable, nor is the work more monotonous if the nurse takes an interest in it, for no two cases are precisely alike. It is new to learn that the intention of offering the most self-denying service to fellow-creatures denotes an unwholesome mind; if such is the case, it is to be feared that many nurses, both hospital and mental, would be in danger of certification. Admitted that pecuniary gain does exert a great influence on the choice of a profession, it is suggested that the predominant factor which turns a woman towards nursing, and incidentally which makes her a good nurse, is a desire to help those who cannot, from either bodily or mental disorder, help themselves, for without this factor, those essential attributes of a nurse, thoroughness in work, a strict sense of obedience, duty toward her hospital and her profession, sympathy and untiring help for her patients, would be found wanting.

Finally, regarding the question of social standing, it is deplorable that controversy should arise. If pure thoughts and high ideals, altruism, an unimpeachable moral character and calm self-control constitute, as they do, the true lady, then there are many nurses in mental hospitals who are every inch of them ladies, even though born of humble parents. Surely, in a noble profession, the highest calling of women, there should be no dissension over such matters. If any hospital-trained nurse refuses to work alongside her sister trained in mental nursing, or refuses her help merely because she is of humble birth, then all that can be said is that the hospital nurse is showing a most unpatriotic spirit at a time when all should unite in a common cause to tend, when called on, those who fight or are unable to fight for England, working cheerfully and amicably side by side and remembering that a nurse is a nurse whatever her social standing. If this is so, then such a nurse is an exception to the majority of Britons, and ceases by her own act to have the right to be called a lady.—Yours, etc.,

A Supporter of "Justice."

[Our correspondent is refuting what was never asserted. We have never said that a mental nurse is ignorant, or that she is a maid, or that her training is a farce. She may be learned, she may be a married woman and a joyful mother of children, and she may have had an excellent training in nursing cases of mental disease; but she is not a competent surgical or medical nurse unless, in addition to her training in an asylum, she has been trained in a general hospital. Our correspondent's assumption that an unwholesome mind means an insane mind shows that her own training in mental nursing has not been sufficient to teach her what insanity is. It is inexpedient and unwise to discuss any further the relative social positions of mental and other nurses. The facts are notorious, and cannot be altered by denial or discussion.—Ed. The Hospital.]

